# Effect of Kinesin-5 Tail Domain on Motor Dynamics for Antiparallel Microtubule Sliding

**DOI:** 10.3390/ijms22157857

**Published:** 2021-07-23

**Authors:** Yuying Liu, Yao Wang, Pengye Wang, Ping Xie

**Affiliations:** 1 College of Science, China Agricultural University, Beijing 100083, China; liuyuying@cau.edu.cn; 2 College of Engineering, China Agricultural University, Beijing 100083, China; 2018307140110@cau.edu.cn; 3 Key Laboratory of Soft Matter Physics, Institute of Physics, Chinese Academy of Sciences, Beijing 100190, China; pywang@aphy.iphy.ac.cn

**Keywords:** kinesin-5, antiparallel microtubule sliding, tail domain, molecular motor, catch-bond

## Abstract

Kinesin-5 motor consists of two pairs of heads and tail domains, which are situated at the opposite ends of a common stalk. The two pairs of heads can bind to two antiparallel microtubules (MTs) and move on the two MTs independently towards the plus ends, sliding apart the two MTs, which is responsible for chromosome segregation during mitosis. Prior experimental data showed that the tails of kinesin-5 Eg5 can modulate the dynamics of single motors and are critical for multiple motors to generate high steady forces to slide apart two antiparallel MTs. To understand the molecular mechanism of the tails modulating the ability of Eg5 motors, based on our proposed model the dynamics of the single Eg5 with the tails and that without the tails moving on single MTs is studied analytically and compared. Furthermore, the dynamics of antiparallel MT sliding by multiple Eg5 motors with the tails and that without the tails is studied numerically and compared. Both the analytical results for single motors and the numerical results for multiple motors are consistent with the available experimental data.

## 1. Introduction

Kinesin-5 molecular motors constitute a subfamily of the large kinesin superfamily that can perform various functions in cells such as cargo transport, chromosome segregation, spindle assembly, cytoskeletal organization, etc., via interacting with microtubules (MTs) [1,2,3]. A kinesin-5 motor is a homotetramer, consisting of two pairs of N-terminal motor domains (or heads) and two pairs of C-terminal tail domains, which are situated at the opposite ends of a common 60-nm-long rod-like stalk [4,5] (see Figure S1 in Supplementary Materials). Each head is connected to the stalk via its flexible neck linker (NL) of 18 residues. One pair of heads of the tetramer can bind to one MT and the other pair can bind to another antiparallel MT, crosslinking the two MTs. By making use of the chemical energy released from ATP hydrolysis the two pairs of heads can move on the two antiparallel MTs independently, sliding apart the two MTs, responsible for chromosome segregation during mitosis [6,7,8,9]. Except for some fungal kinesin-5 motors such as *S. cerevisiae* Cin8 and Kip1 and *S. pombe* Cut7 that can move processively towards either plus or minus end of MT, which depends on the experimental conditions [9,10], most kinesin-5 motors such as vertebrate Eg5 and *Drosophila* Klp61F move exclusively towards the plus end of MT [6,7], like kinesin-1 dimer. In this work, we focus only on vertebrate Eg5.

To understand the detailed mechanism and dynamics of the generation of forces to slide apart MTs by Eg5 molecular motors, the load dependences of velocity and run length of the single Eg5 without the C-terminal tail domain (abbreviated as Eg5-ΔTail) moving on the single MT were determined experimentally [11,12]. The load dependence of velocity of the single full-length Eg5 containing the tail domain (abbreviated as FL-Eg5) moving on the single MT was studied well and the MT-sliding forces generated by multiple FL-Eg5 motors were also studied in detail [13]. To understand the role of the tail domain in MT sliding, the ATPase activities of Eg5-ΔTail and FL-Eg5 motors were studied biochemically, the interactions between the tail domain and head of Eg5 in different nucleotide states were studied biochemically and structurally, and the forces generated by multiple Eg5-ΔTail and FL-Eg5 motors were measured using optical trappings [14]. Interestingly, it was found that the tail domains decrease MT-stimulated ATPase rate of the motor by specifically engaging heads in the nucleotide-free and ADP states, and the FL-Eg5 motors can generate high steady forces whereas Eg5-ΔTail motors cannot [14].

While the experimental data on the load dependences of velocity and run length for the single Eg5-ΔTail moving on the single MT were explained quantitatively using numerical simulations [15], the experimental data on the load dependence of velocity for the single FL-Eg5 moving on the single MT have not been explained theoretically. The mechanism of the modulation of the tail domain on the dynamics of the single Eg5 is unclear. The effect of the tail domain on the load dependences of run length and dissociation rate of the single Eg5 is unknown. The mechanism of the tail domain influencing the MT-sliding force generated by Eg5 motors is unclear. The purpose of this work is to address the above unclear issues. To this end, a model for the processive stepping of the single FL-Eg5 on the single MT is proposed on the basis of the previously proposed model for the processive stepping of the single Eg5-ΔTail on the single MT and the available experimental data on the interaction between the tail and head of Eg5. With the model, the load dependences of velocity, ATPase rate, run length and dissociation rate for the single FL-Eg5 moving on the single MT are studied analytically, which are compared with the corresponding results for the single Eg5-ΔTail. Furthermore, the MT-sliding force generated by multiple Eg5-ΔTail and that by FL-Eg5 motors were studied numerically and compared. The analytical and numerical results are consistent with the available experimental data.

## 2. Results and Discussion

### 2.1. Dynamics of Single Eg5-ΔTail Moving on Single MT

**Figure 1:**
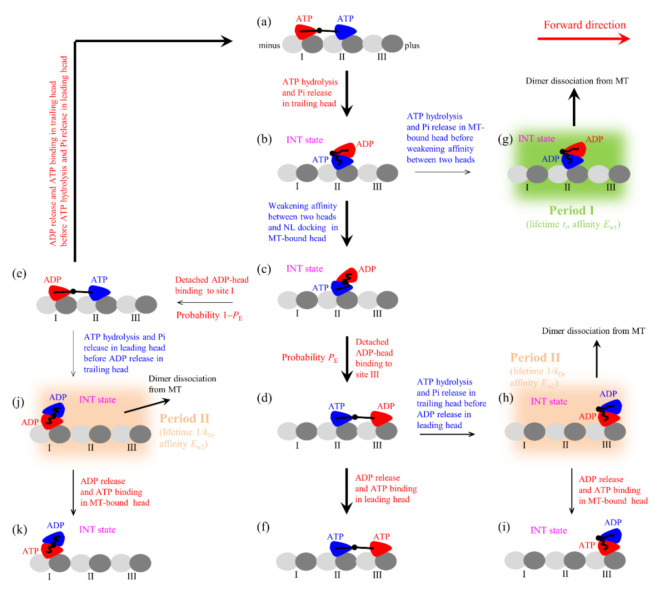
Model of the single Eg5-ΔTail moving on the single MT filament at saturating ATP and the occurrence of the weak-affinity periods (Period I and Period II) when the motor binds weakly to MT (see Methods for detailed descriptions). (**a**–**k**) The chemomechanical coupling pathway. For clarity, only the pair of heads that moves on the MT filament is drawn. In addition, only the transitions following ATP hydrolysis and Pi release in the trailing heads are shown while the similar transitions following ATP hydrolysis and Pi release in the leading heads are not shown. The thickness of the arrow represents the magnitude of the transition rate or probability under no load.

**Figure 2:**
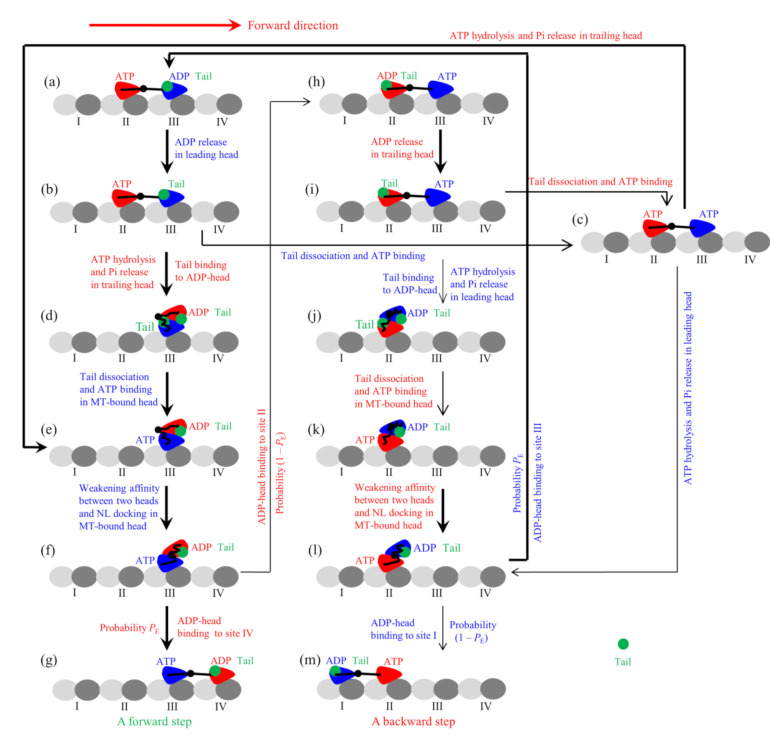
Model of the single FL-Eg5 moving on the single MT filament at saturating ATP (see Methods for detailed descriptions). (**a**–**m**) The chemomechanical coupling pathway. For clarity, only the pair of heads that moves on the MT filament is drawn, and the tail unbound to the head is not drawn. The occurrence of the weak-affinity periods (Period I and Period II) when the motor binds weakly to MT is not shown. The thickness of the arrow represents the magnitude of the transition rate or probability under no load.

In this section, on the basis of the model for the chemomechanical coupling of Eg5-ΔTail (Figure 1, see Methods for detailed descriptions), we study the dynamics of the single Eg5-ΔTail motor moving on the single MT. Consider a load *F* on the stalk of the motor, with *F* < 0 (*F* > 0) being the backward (forward) load. As derived before [16], the load dependence of effective probability *P*_E_ (defined in Figure 1) has the form

(1) $$P_{E}=\frac{\exp\left(\beta E_{D}\right)\exp\left(\beta Fd^{\left(+\right)}\right)}{\exp\left(\beta E_{D}\right)\exp\left(\beta Fd^{\left(+\right)}\right)+1}$$

where *E*_D_ is the energy change associated with both the NL docking and the conformational change of the head induced by ATP binding, *d*^(+)^ is characteristic distance for the movement of the detached head from INT position to either the forward or backward binding site on MT, and $\beta^{-1}=k_{\mathrm{Boltzman}}T$ being the thermal energy. Note that in the pathway for FL-Eg5 (Figure 2, see Methods for detailed descriptions), which is modified from that for Eg5-ΔTail, *P*_E_ has the same form.

Before presenting equations for velocity and run length, let us define rate constants of the ATPase activity and NL docking. We denote by *k*^(+)^ the rate of ATP transition to ADP in the head with the forward NL orientation (e.g., the trailing head) and by *k*^(−)^ the rate of ATP transition to ADP in the head without the forward NL orientation (e.g., the leading head). Rates *k*^(+)^ and *k*^(−)^ are independent of the force on the NLs, which are consistent with the available experimental data (see, e.g., [17,18,19] for detailed discussion). We denote by *k*_D_ the rate of ADP release from the head bound to MT, which for simplicity is treated here to be independent of NL direction and force on NL. Since both the rate of weakening the affinity of the MT-bound ATP-head to the detached ADP-head and the rate of NL docking of the MT-bound ATP-head are determined by the rate of the large conformational change of the ATP-head, the three rates have approximately the same values. Thus, we use *k*_NL_ to represent both the rate of NL docking and that of weakening between the two heads.

Considering that *k*_NL_ >> *k*^(+)^ (see later) and the weak MT-binding periods (Period I and Period II) occur occasionally and when it occurs the motor has a large probability to dissociate from MT, the overall ATPase rates of trailing and leading heads can be approximately calculated with [16]

(2) $$k_{T}=P_{E}k^{\left(+\right)}+\left(1-P_{E}\right){\left(\frac{1}{k_{D}}+\frac{1}{k^{\left(+\right)}}\right)}^{-1}$$

(3) $$k_{L}=P_{E}{\left(\frac{1}{k_{D}}+\frac{1}{k^{\left(-\right)}}\right)}^{-1}+\left(1-P_{E}\right)k^{\left(-\right)}$$

The total ATPase rate of the motor is *k*_T_ + *k*_L_. The overall forward stepping rate of the motor is *P*_E_*k*_T_ and the overall backward stepping rate is (1 − *P*_E_)*k*_L_. The velocity of the motor can then be written as

(4) $$v=\left[P_{E}k_{T}-\left(1-P_{E}\right)k_{L}\right]d$$

where *d* = 8.2 nm is the step size.

The occurrence probability of Period I, *P*_I_, can be calculated with [16,20]

(5) $$P_{I}=\frac{k^{\left(-\right)}}{k_{\mathrm{NL}}+k^{\left(-\right)}},\;\mathrm{when}\;F\;\leq \;0,$$

(6) $$P_{I}=\frac{k^{\left(+\right)}}{k_{\mathrm{NL}}+k^{\left(+\right)}},\;\mathrm{when}\;F\;\geq \;4\;\mathrm{pN}$$

where it is considered that under no or a backward load (*F* ≤ 0) on the stalk the NL of the MT-bound head in INT state before NL docking is not in the forward orientation, with the rate of ATP transition to ADP equal to *k*^(−)^, while under a forward load larger than or equal to 4 pN (*F* ≥ 4 pN) the NL of the MT-bound head in INT state before NL docking is driven to be in the forward orientation, with the rate of ATP transition to ADP equal to *k*^(+)^. Under the forward load smaller than 4 pN, the following phenomenological form can be adopted for the rate of ATP transition to ADP in the MT-bound head [20]

(7) $$k=k^{\left(-\right)}+\frac{k^{\left(+\right)}-k^{\left(-\right)}}{4}F,\;\mathrm{when}\;0\;<\;F\;<\;4\;\mathrm{pN},$$

where *k* = *k*^(−)^ when *F* = 0 and *k* = *k*^(+)^ when *F* = 4 pN. The occurrence probability of Period I can then be calculated with

(8) $$P_{I}=\frac{k}{k_{\mathrm{NL}}+k},\;\mathrm{when}\;0\;<\;F\;<\;4\;\mathrm{pN}.$$

In Period I, since *E*_w1_ is very small the motor can dissociate from MT with a nearly 100% probability even under no load, giving the dissociation probability in Period I *P*_dI_ ≈ 1 under any load.

The occurrence probability of Period II, *P*_II_, can be calculated with [16]

(9) $$P_{\mathrm{II}}=P_{E}\frac{k^{\left(+\right)}}{k^{\left(+\right)}+k_{D}}+\left(1-P_{E}\right)\frac{k^{\left(-\right)}}{k^{\left(-\right)}+k_{D}}$$

The dissociation probability, *P*_dII_, in Period II can be calculated with

(10) $$P_{\mathrm{dII}}=\frac{k_{\mathrm{dII}}}{k_{\mathrm{dII}}+k_{D}}$$

(11) $$k_{\mathrm{dII}}=k_{w0}\exp\left(\beta \left|F\right|\delta_{w}\right)$$

where *k*_dII_ is the dissociation rate during Period II, with *k*_w0_ being the dissociation rate under no load and $\delta_{w}$ being the distance parameter for dissociation. To be consistent with the Debye length of about 1 nm in solution, we take $\delta_{w}$ = 1 nm.

For approximation, in this work we neglect the dissociation of kinesin in the strong MT-binding state. Thus, the dissociation rate can be calculated with [16,20]

(12) $$\varepsilon =k_{T}P_{I}P_{\mathrm{dI}}+\left(k_{T}+k_{L}\right)P_{\mathrm{II}}P_{\mathrm{dII}}$$

where *k*_T_ is calculated by Equation (2), *k*_L_ is calculated by Equation (3), *P*_I_ is calculated by Equations (5)–(8), *P*_dI_ = 1, *P*_II_ is calculated by Equation (9), and *P*_dII_ is calculated by Equations (10) and (11). The run length can then be calculated with

(13) $$L=\frac{v}{\varepsilon }$$

where *v* is calculated with Equation (4) and ε is calculated with Equation (12).

Before presenting our theoretical results using the above equations, we firstly discuss the choice of the parameter values. The biochemical data for kinesin-1 head showed that without NL the ATPase rate of the head is reduced largely while the ADP release rate is not affected [21], implying that the interaction of NL in the forward (or docked) direction enhances the rate of ATP transition to ADP. Thus, we take the rate constant of ATP transition to ADP in Eg5 head with the NL in the forward direction being also larger than that in the head without in the forward direction, with *k*^(−)^ = *k*^(+)^/15. The velocity at the large forward load is determined mainly by *k*^(+)^. To be consistent with the single-molecule data of Valentine et al. [11] at the large forward load, we take *k*^(+)^ = 12.8 $s^{-1}$. Note that the above value of *k*^(+)^ is close to that determined biochemically [22]. Since the curve form of velocity versus load is determined mainly by *E*_D_ and *d*^(+)^, to be consistent with the single-molecule data of Valentine et al. [11], we adjust values of *E*_D_ and *d*^(+)^, with *E*_D_ = 2.5 *k*_B_*T* and *d*^(+)^ = 2.2 nm. Note that this value of *E*_D_ = 2.5 *k*_B_*T* is consistent with the experimentally measured free energy change associated with NL docking for kinesin-1 head to be smaller than 1 *k*_B_*T* [23] and the atomistic MD simulated free energy change associated with the large conformational change to be only about 1.7 *k*_B_*T* for kinesin-1 head [24]. Since the run length for Eg5 under no load is determined mainly by *k*_NL_, *k*_D_ and *k*_w0_ besides the unloaded velocity, to be consistent with the single-molecule data of Valentine et al. [12] for the unloaded run length, we adjust values of *k*_NL_, *k*_D_ and *k*_w0_, with *k*_NL_ = 200 $s^{-1}$, *k*_D_ = 50 $s^{-1}$ and *k*_w0_ = 20 $s^{-1}$. The above value of *k*_D_ is similar to that measured biochemically for Eg5 [22]. The above value of *k*_NL_ for Eg5 (200 $s^{-1}$) is much smaller than that for kinesin-1 (1500 $s^{-1}$) determined before [16,20], which is also consistent with the biochemical results for Eg5 and kinesin-1 [22,25]. For clarity, the parameter values are listed in Table 1.

**Figure 3:**
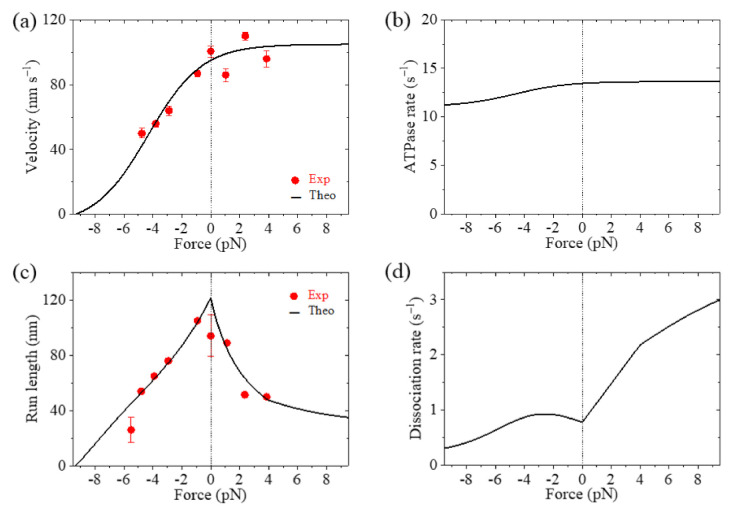
Results for dynamics of the single Eg5-ΔTail moving on the single MT. Lines are theoretical results and dots are experimental data measured by Valentine et al. [11,12]. (**a**) Velocity versus load. (**b**) ATPase rate versus load. (**c**) Run length versus load. (**d**) Dissociation rate versus load.

The theoretical results of velocity, ATPase rate, run length and dissociation rate versus load for Eg5-ΔTail are shown in Figure 3a–d, respectively, where the available single-molecule data [11,12] are also shown. It is seen that the theoretical results for the velocity and run length versus load are consistent with the experimental data (Figure 3a,c). The results show that the stall force, at which the velocity and run length are equal to zero, is about 9.3 pN (Figure 3a,c). From Figure 3b, it is seen that the ATPase rate of the motor changes only slightly with the increase in the magnitude of backward load and is kept almost unchanged with the forward load. This feature is consistent with the experimental data for kinesin-1 dimer showing that the ATPase rate is insensitive to the variation of the NL length (equivalent to the variation of the internal force) [26]. From Figure 3d, it is seen that the dissociation rate increases rapidly with the increase of the forward load, while the dissociation rate increases slightly with the increase in the magnitude of the backward load smaller than 2.6 pN and then decreases slightly with the further increase in the magnitude of the backward load.

### 2.2. Dynamics of Single FL-Eg5 Moving on Single MT

In this section, on the basis of the chemomechanical coupling pathway for FL-Eg5 (Figure 2, see Methods for detailed descriptions), which is modified from that for Eg5-ΔTail by considering the nucleotide-dependent interaction between the tail and head, we study the dynamics of the single FL-Eg5 motor moving on the single MT. As discussed in the model for FL-Eg5 (see Methods), since the tail has a near-zero rate to release from the ADP-head, its release from the ADP-head can be neglected. The tail has a low rate to release from ϕ-head, which is denoted by *k*_r_. Only after the release of the tail from ϕ-head can ATP bind to the ϕ-head, implying that at saturating ATP after the release of the tail from ϕ-head ATP can bind immediately. In addition, we argue here that the strong interaction between the tail and ADP-head induces the conformational change of the head, increasing the weak affinity *E*_w2_ of the ADP-head to MT and affecting the interaction potential of the ADP-head with MT. Thus, the binding of the tail to ADP-head could also affect *d*^(+)^.

From the pathway (Figure 2), it is noted that both the rate for the fraction of transitions from Figure 2a to b to c to e and that for the fraction of transitions from Figure 2a to b to d are equal to *k*^(+)^. The proportion of the fraction of transitions from Figure 2a to b to d (i.e., the proportion of the fraction of the transitions where the tail has not been released in Figure 2d) in the total transitions from Figure 2a to e can be approximately written as $P_{0T}=\left(1/k_{D}+1/k_{r}\right)/\left(1/k_{D}+1/k_{r}+1/k^{\left(+\right)}\right)$. The rate of transition from Figure 2d to e is *k*_r_. Thus, considering *k*_NL_ >> *k*^(+)^ the average rate of the total transitions from Figure 2a to e to f can be approximately calculated with ${\left(1/k^{\left(+\right)}+P_{0T}1/k_{r}\right)}^{-1}$. Considering *k*_NL_ >> *k*^(+)^ the rate for the transition from Figure 2h to i to c to e to f can be approximately written as ${\left(1/k_{D}+1/k_{r}+1/k^{\left(+\right)}\right)}^{-1}$. Since during the processive motion the state of the motor with the trailing head in ATP state and the leading head in ADP state (Figure 2a) occurs with probability *P*_E_ while the state of the motor with the trailing head in ADP state and the leading head in ATP state (Figure 2h) occurs with probability 1 − *P*_E_, the overall ATPase rate of the trailing head can then be approximately written as

(14) $$k_{T}=P_{E}{\left(\frac{1}{k^{\left(+\right)}}+P_{0T}\frac{1}{k_{r}}\right)}^{-1}+\left(1-P_{E}\right){\left(\frac{1}{k_{D}}+\frac{1}{k_{r}}+\frac{1}{k^{\left(+\right)}}\right)}^{-1}$$

where $P_{0T}=\left(1/k_{D}+1/k_{r}\right)/\left(1/k_{D}+1/k_{r}+1/k^{\left(+\right)}\right)$ (see above).

Similarly, considering *k*_NL_ >> *k*^(−)^ the overall ATPase rates of the leading head can then be approximately written as

(15) $$k_{L}=P_{E}{\left(\frac{1}{k_{D}}+\frac{1}{k_{r}}+\frac{1}{k^{\left(-\right)}}\right)}^{-1}+\left(1-P_{E}\right){\left(\frac{1}{k^{\left(-\right)}}+P_{0L}\frac{1}{k_{r}}\right)}^{-1}$$

where $P_{0L}=\left(1/k_{D}+1/k_{r}\right)/\left(1/k_{D}+1/k_{r}+1/k^{\left(-\right)}\right)$ (see above).

From the pathway (Figure 2), it is noted that the velocity can still be written in the form of Equation (4), where *P*_E_ is still calculated by Equation (1) and *k*_T_ and *k*_L_ are calculated by Equations (14) and (15), respectively. The occurrence probability of Period I, *P*_I_, can also be calculated by Equations (5)–(8). The dissociation probability in Period I is still *P*_dI_ ≈ 1, for upon Pi release the time of Period I is so short (in the order of 10 μs) that the tail cannot bind to the ADP-head during the period. The occurrence probability of Period II, *P*_II_, can still be calculated by Equation (9), and the dissociation probability *P*_dII_ in Period II can also be calculated by Equations (10) and (11). For simplicity, we neglect the very small occurrence probability of state of Figure 2j, from which Period I can also occur occasionally. The dissociation rate can then still be expressed in the form of Equation (12) and the run length can still be written in the form of Equation (13).

As discussed above, for FL-Eg5, except for parameters *k*_w0_, *d*^(+)^ and *k*_r_ other parameters have the same values as those for Eg5-ΔTail. Here, we take *k*_w0_ = 5 $s^{-1}$ for FL-Eg5 (Table 1), which is 4-fold smaller than that for Eg5-ΔTail. For FL-Eg5, besides taking *d*^(+)^ = 2.2 nm, which is the same as that for Eg5-ΔTail, we also take *d*^(+)^ = 8.2 nm, which is equal to *d*. To be consistent with the experimental data of the velocity under near-zero load measured by Shimamoto et al. [13], we adjust value of *k*_r_, with *k*_r_ = 4.6 $s^{-1}$ (Table 1).

**Figure 4:**
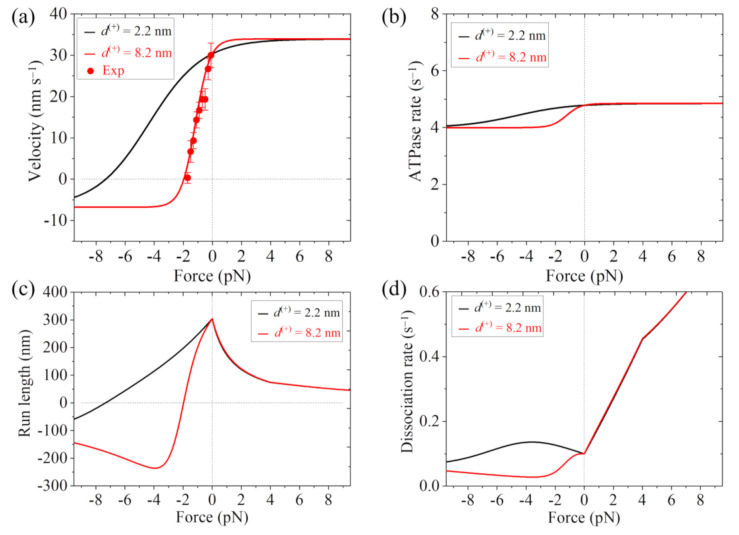
Results for dynamics of the single FL-Eg5 moving on the single MT. Lines are theoretical results and dots are experimental data measured by Shimamoto et al. [13]. (**a**) Velocity versus load. (**b**) ATPase rate versus load. (**c**) Run length versus load. (**d**) Dissociation rate versus load.

In Figure 4a–d, we show the theoretical results of velocity, ATPase rate, run length and dissociation rate versus load, respectively, for FL-Eg5, where the experimental data of Shimamoto et al. [13] are also shown. It is seen that the theoretical results of the velocity versus load with *d*^(+)^ = 8.2 nm are in good agreement with the experimental data (Figure 4a). The results show that with *d*^(+)^ = 2.2 nm the stall force for FL-Eg5 is about 7.3 pN (Figure 4a,c), which is smaller than 9.3 pN for Eg5-ΔTail with the same *d*^(+)^ = 2.2 nm (Figure 3a,c). With *d*^(+)^ = 8.2 nm the stall force for FL-Eg5 is only about 2 pN (Figure 4a,c). Comparing Figure 3b and Figure 4b it is seen that with the tail the ATPase rate of Eg5 is reduced by more than 2-fold, which is consistent with the experimental data of Bodrug et al. [14]. Comparing Figure 3c and Figure 4c it is seen that for FL-Eg5 the run length under no load is more than 2-fold larger than that for Eg5-ΔTail, although the velocity for the former is smaller than that for the latter (Figure 3a and Figure 4a), which is consistent with the experimental data of Bodrug et al. [14]. From Figure 4d, it is seen that under the forward load the dissociation rate versus load for FL-Eg5 shows the similar feature to that for Eg5-ΔTail. Under the backward load, for FL-Eg5 with *d*^(+)^ = 2.2 nm the dissociation rate increases slightly with the increase in the magnitude of the load smaller than 3.6 pN and then decreases slightly with the further increase in the magnitude of the backward load, which is similar to that for Eg5-ΔTail. For FL-Eg5 with *d*^(+)^ = 2.2 nm the dissociation rate under the backward load around the stall force is about 3.3-fold smaller than that for Eg5-ΔTail, similar to the value of *k*_w0_ for FL-Eg5 relative to that for Eg5-ΔTail. Interestingly, from Figure 4d, it is seen that for FL-Eg5 with *d*^(+)^ = 8.2 nm the dissociation rate decreases monotonically and largely with the increase in the magnitude of the backward load (behaving as catch-bond characteristic) before the stall force of about 2 pN, which is in sharp contrast to that for Eg5-ΔTail and that for FL-Eg5 with *d*^(+)^ = 2.2 nm. More interestingly, by comparing Figure 3d with Figure 4d, it is seen that for FL-Eg5 with *d*^(+)^ = 8.2 nm the dissociation rate under the backward load around the stall force is much (about 10-fold) smaller than that for Eg5-ΔTail, although *k*_w0_ for FL-Eg5 is only 4-fold smaller than that for Eg5-ΔTail. Since the low dissociation rate under the backward load around the stall force is critical for exerting long-time force to slide apart two antiparallel MTs by the motor whereas the unloaded velocity is insignificant for exerting force, the above results give an explanation of why Eg5 motor with the tail has the smaller velocity and smaller stall force or larger *d*^(+)^ than without the tail.

### 2.3. Dynamics of Sliding Apart Two Antiparallel MTs by Multiple Motors

In this section we study numerically the force generated by multiple kinesin-5 motors to slide apart two antiparallel MTs. The numerical simulation procedure is described as follows (refer to Figure S2 in Supplementary Materials).

To calculate the force, we fix the two antiparallel MT filaments. A pair of kinesin-5 heads at one end of the stalk binds to one of the two antiparallel MT filaments with rate *k*_a_, where *k*_a_ is proportional to the concentration of kinesin-5 motors in solution and the MT overlapping length. Then, the other pair of heads at the opposite end of the stalk can bind to the antiparallel MT filament with rate $\mu_{5}$. Note that when only one pair of heads is bound to MT, the other pair of heads at the opposite ends of the stalk is considered to be in the same position along the *x* direction (parallel to the MT filament). After two pairs of heads binding to MTs, the two pairs move independently on the MTs. Each pair of heads takes a forward (plus-end) step with rate *k*_F_ = *P*_E_*k*_T_, takes a backward (minus-end) step with rate *k*_B_ = (1 − *P*_E_)*k*_L_, and detaches from MT with rate ε. After one pair of heads detaching from MT, the detached pair of heads moves immediately to the position of the other MT-bound pair of heads along the *x* direction. The MT-bound pair of heads can detach from the MT still with rate ε and the detached pair of heads can rebind to the former MT with rate $\mu_{5}$. (Since when only one pair of heads is bound to MT no force can be generated on MTs, the movement of the pair of heads on MT is not needed to consider). If the detachment of the MT-bound pairs of heads occurs before the rebinding of the detached pair of heads to MT, the tetramer is dissociated into solution. When one kinesin-5 tetramer (termed as, e.g., the *i*th motor) is bound to MTs in the overlap, denoting by $x_{1}^{\left(i\right)}$ the center-of-mass position of one pair of heads in the MT with the plus end in the *x* direction and by $x_{2}^{\left(i\right)}$ the center-of-mass position of the other pair of heads in the MT with the plus end in the − *x* direction, the force generated by the *i*th motor to slide apart MTs can be calculated with $F_{\mathrm{MT}}^{\left(i\right)}=K_{5}\left(x_{1}^{\left(i\right)}-x_{2}^{\left(i\right)}\right)$, where *K*_5_ is the stretching elastic coefficient of kinesin-5 stalk. When *N* kinesin-5 motors are bound to the overlapping MTs, the total force generated can be calculated by $F_{\mathrm{MT}}=\sum_{i=1}^{N}F_{\mathrm{MT}}^{\left(i\right)}$. For simplicity of analysis, here we do not consider the interaction between any two kinesin-5 motors. The above procedure is simulated with Monte-Carlo algorithm (see Section S1 in Supplementary Materials), as done before [27,28].

The forward stepping rate *k*_F_ = *P*_E_*k*_T_, backward stepping rate *k*_B_ = (1 − *P*_E_)*k*_L_ and dissociation rate ε for one pair of heads of Eg5-ΔTail and those of FL-Eg5 are determined above. We take both Eg5-ΔTail and FL-Eg5 having the same binding rate *k*_a_, which is taken as a variable parameter in this work. The rebinding rate $\mu_{5}$ and the elastic coefficient of the stalk for Eg5-ΔTail and those for FL-Eg5 are determined as follows.

Since in the system of cargo transported by multiple kinesin-1 motors the rebinding rate of one detached kinesin-1 motor to MT (denoted by $\mu_{1}$) is available in the literature (see below), the rebinding rate $\mu_{5}$ can be approximately determined from $\mu_{1}$, as described as follows. Consider that a Brownian particle is connected to a fixed point via a stalk of length *l* and the stalk can rotate in one-dimensional potential $V\left(\theta \right)=k\theta^{2}/2$, where θ is the rotation angle and *k* is constant. Thus, the position of the Brownian particle is x=lθ. The probability of the stalk with rotation angle θ has the form

(16) $$P\left(\theta \right)={\left(\frac{k}{2\pi k_{B}T}\right)}^{1/2}\exp\left(-\frac{k\theta^{2}}{2k_{B}T}\right)$$

The probability of the position of the Brownian particle can then be written as

(17) $$P\left(x\right)={\left(\frac{k}{2\pi k_{B}T}\right)}^{1/2}\exp\left(-\frac{kx^{2}}{2l^{2}k_{B}T}\right)$$

As it is known, in solution when the distance between a particle and its partner is larger than the Debye length *a* = 1 nm, nearly no interaction between them exists. Thus, the binding rate of the Brownian particle to its partner that is kept in the position of distance *l* away from the point to which the stalk of the Brownian particle is connected can be approximately calculated with $\mu =C\int_{-a}^{a}P\left(x\right)dx$, where *C* is a constant that is inversely proportional to the dissociation rate of the particle from its partner. Let *l* = *l*_1_ and *l* = *l*_5_ be the stalk lengths of kinesin-1 and kinesin-5 motors, respectively. Then, the ratio of $\mu_{5}$ to $\mu_{1}$ can be calculated by

(18) $$\frac{\mu_{5}}{\mu_{1}}=\frac{k_{w0}^{\left(1\right)}}{k_{w0}^{\left(5\right)}}\frac{\int_{-a}^{a}\exp\left(-\frac{kx^{2}}{2{\left(l_{5}\right)}^{2}k_{B}T}\right)dx}{\int_{-a}^{a}\exp\left(-\frac{kx^{2}}{2{\left(l_{1}\right)}^{2}k_{B}T}\right)dx}$$

where $k_{w0}^{\left(5\right)}$ represents *k*_w0_ for kinesin-5 head in ADP state and $k_{w0}^{\left(1\right)}$ represents *k*_w0_ for kinesin-1 head in ADP state. As determined above, $k_{w0}^{\left(5\right)}$ = 20 $s^{-1}$ for Eg5-ΔTail and $k_{w0}^{\left(5\right)}$ = 5 $s^{-1}$ for FL-Eg5 (see Table 1). As determined before, $k_{w0}^{\left(1\right)}$ = 5 $s^{-1}$ [16]. The available data gave *l*_5_ = 61.3 nm [4]. As done before [29], we take *l*_1_ = 35 nm. We have checked that varying values of *l*_5_ and *l*_1_ has nearly no effect on the results presented in this work. As shown experimentally before [30] and used in a widespread manner [31,32], we take $\mu_{1}$ = 5 $s^{-1}$. With above parameter values and from Equation (18) we obtain $\mu_{5}$ ≈ 1.25 $s^{-1}$ for Eg5-ΔTail and $\mu_{5}$ ≈ 5 $s^{-1}$ for FL-Eg5, which are nearly independent of the value of *k*. Thus, in our numerical simulations we take $\mu_{5}$ = 1.25 $s^{-1}$ and 5 $s^{-1}$ for Eg5-ΔTail and FL-Eg5, respectively.

As done before in [27,28,29], it is considered that the kinesin stalk can be stretched elastically. Since both Eg5-ΔTail and FL-Eg5 having the same length, they have the same stretching elastic coefficient, with the value being determined as follows. The available experimental data showed that the elastic coefficient for kinesin-1 stalk is *K*_1_ = 0.3 pN/nm [33]. Thus, the elastic coefficient for kinesin-5 stalk can be estimated as $K_{5}=K_{1}l_{1}/l_{5}$ = 0.17 pN/nm. We take this value of *K*_5_ in our simulations. However, we have checked that varying value of *K*_5_ has nearly no effect on the results presented in this work.

**Figure 5:**
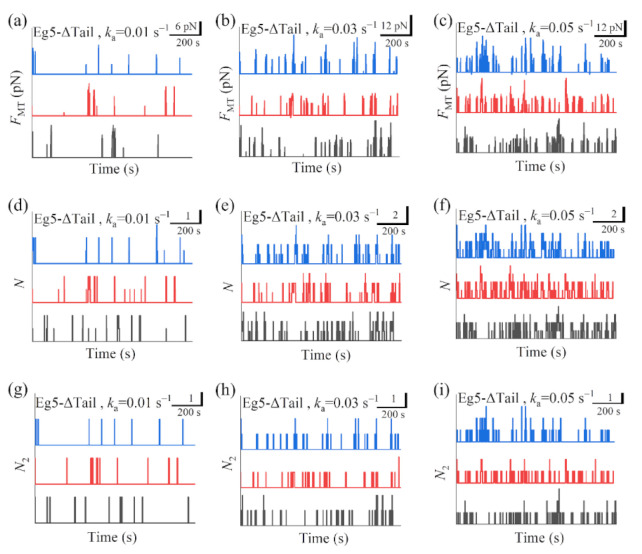
Some results for dynamics of sliding apart two antiparallel MTs by multiple Eg5-ΔTail motors. (**a**–**c**) Temporal evolution of the generated MT-sliding force *F*_MT_ with different values of motor-binding rate *k*_a_. (**d**–**f**) Temporal evolution of the total number of the motors, *N*, bound in the MT overlap with different values of motor-binding rate *k*_a_. (**g**–**i**) Temporal evolution of the number of the motors, *N*_2_, with two pairs of heads bound to the MTs in the overlap with different values of motor-binding rate *k*_a_. In all panels *t* = 0 corresponds to the moment when only one motor binds to the overlapping MTs. The three curves (black, red and blue) in each panel correspond to three independent realizations in the simulation.

**Figure 6:**
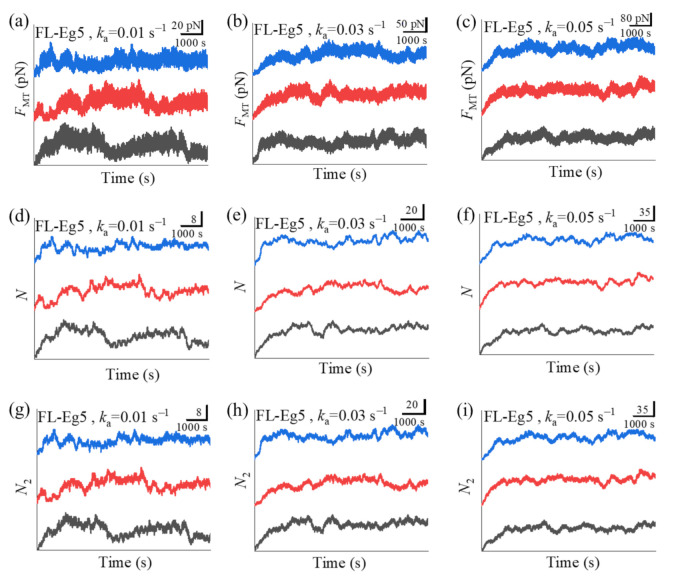
Some results for dynamics of sliding apart two antiparallel MTs by multiple FL-Eg5 motors. (**a**–**c**) Temporal evolution of the generated MT-sliding force *F*_MT_ with different values of motor-binding rate *k*_a_. (**d**–**f**) Temporal evolution of the total number of the motors, *N*, bound in the MT overlap with different values of motor-binding rate *k*_a_. (**g**–**i**) Temporal evolution of the number of the motors, *N*_2_, with two pairs of heads bound to the MTs in the overlap with different values of motor-binding rate *k*_a_. In all panels *t* = 0 corresponds to the moment when only one motor binds to the overlapping MTs. The three curves (black, red and blue) in each panel correspond to three independent realizations in the simulation.

In Figure 5a–c we show some simulated results for the temporal evolution of the force *F*_MT_ generated by multiple Eg5-ΔTail motors under different values of motor-binding rate *k*_a_, where *t* = 0 corresponds to the moment when only one motor binds to the overlapping MTs. The corresponding results for the total number of Eg5-ΔTail motors, *N*, in the MT overlap are shown in Figure 5d–f. The corresponding results for the number of Eg5-ΔTail motors with two pairs of heads bound to the MTs, *N*_2_, are shown in Figure 5g–i (noting that *N*_2_ corresponds to the effective number of the motors that can generate the force). For comparison between the case of Eg5-ΔTail motors and that of FL-Eg5 motors, in Figure 6 we show some simulated results for the temporal evolution of *F*_MT_, *N* and *N*_2_ for the case of FL-Eg5 motors (with *d*^(+)^ = 8.2 nm) under the same values of *k*_a_ as those in Figure 5.

**Figure 7:**
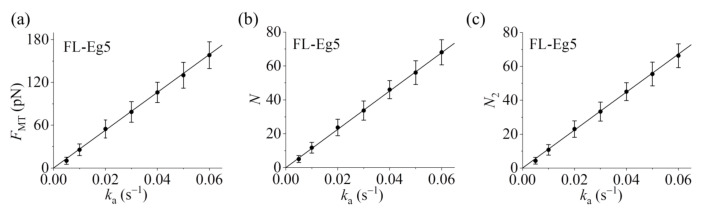
Statistical results for dynamics of sliding apart two antiparallel MTs by multiple FL-Eg5 motors. (**a**) Steady MT-sliding force *F*_MT_ versus motor-binding rate *k*_a_. (**b**) Total steady number of the motors, *N*, bound in the MT overlap versus motor-binding rate *k*_a_. (**c**) Steady number of the motors, *N*_2_, with two pairs of heads bound to the MTs in the overlap versus motor-binding rate *k*_a_. The average values of *F*_MT_, *N* and *N*_2_ (dots) and the corresponding standard deviations (error bars) for a given *k*_a_ are calculated with the three curves shown in Figure 6 after reaching steady states. Lines are linear fits to the numerical data.

Our results show that for the case of Eg5-ΔTail motors no steady force *F*_MT_ can be generated in the range of *k*_a_ from 0.005 $s^{-1}$ to 0.06 $s^{-1}$ (Figure 5a–c). This is because no steady number of Eg5-ΔTail motors bound to MTs in the overlap can be reached (Figure 5d–i). By contrast, our results show that for the case of FL-Eg5 motors the force *F*_MT_ increases gradually until the maximum steady value is reached at any given *k*_a_ in the range from 0.005 $s^{-1}$ to 0.06 $s^{-1}$ (Figure 6a–c), which is due to the gradual increase in the number of FL-Eg5 motors bound to the MTs in the overlap until the maximum steady number is reached (Figure 6d–i). The rather different feature for the force *F*_MT_ generated by FL-Eg5 motors from that by Eg5-ΔTail is due mainly to the dissociation rate for FL-Eg5 before stall force having catch-bond characteristic (see Figure 4d), the dissociation rate around the stall force for the FL-Eg5 being much (about 10-fold) smaller than that for the Eg5-ΔTail (see Figure 3d and Figure 4d) and the rebinding rate for the FL-Eg5 being 4-fold larger than that for the Eg5-ΔTail (see above). By comparing Figure 6d–f with Figure 6g–i it is noted that nearly all FL-Eg5 motors in the overlap are those with two pairs of heads bound to MTs (with *N* only slightly larger than *N*_2_). It is interestingly noted that the simulated curves of *F*_MT_ versus time for FL-Eg5 motors (Figure 6a–c) resemble the experimental data measured by Shimamoto et al. [13] and by Bodrug et al. [14], with both the simulated and experimental results showing that *F*_MT_ increases over time and becomes leveled off to a large steady value. For Eg5-ΔTail motors, the simulated results (Figure 5a–c) are also similar to the experimental results showing that *F*_MT_ only fluctuates around a small value [14]. Moreover, from Figure 6a–c it is seen that the maximum steady force *F*_MT_ generated by FL-Eg5 motors increases linearly with the increase of *k*_a_, which can be also seen clearly from Figure 7 where the maximum steady *F*_MT_, *N* and *N*_2_ versus *k*_a_ are shown. Since *k*_a_ is proportional to the concentration of kinesin-5 tetramers in solution and the MT overlapping length, the results of Figure 7 imply that for a given concentration of kinesin-5 tetramers the maximum steady *F*_MT_, *N* and *N*_2_ increase linearly with the increase of the MT overlapping length. These results are also consistent with the experimental data of Bodrug et al. [14]. Similarly, for a given MT overlapping length the maximum steady *F*_MT_, *N* and *N*_2_ increases linearly with the increase in the concentration of kinesin-5 tetramers, which is consistent with the experimental data of Shimamoto et al. [13].

It is noted that the experimental data of Bodrug et al. [14] indicated that at high concentration of KCl (100 mM) mainly two FL-Eg5 motors can form a cluster while at 25 mM KCl no cluster can be formed. Here, for simplicity, we have not considered the formation of FL-Eg5 clusters in our simulations, which is applicable to the case of low concentration of KCl (closer to physiological conditions). At the high concentration of KCl (100 mM), the formation of clusters could further reduce the dissociation rate of the motors, as proposed before [14], and thus further facilitate the generation of the steady MT-sliding force by FL-Eg5 motors, which will be studied theoretically and numerically in the future.

In addition, we also study numerically the dynamics of Eg5 motors moving within the overlapping MTs (see Section S2 in Supplementary Materials), where one MT is fixed and the other antiparallel MT is free. The numerical results showed that while Eg5-ΔTail shows bi-directional movement with frequent directional reversals FL-Eg5 shows unidirectional movement with infrequent directional reversals (see Section S2 and Figure S3 in Supplementary Materials). These results resemble well the experimental data of Bodrug et al. [14]. The movement of the free MT by multiple FL-Eg5 motors is also simulated, with the velocity being nearly independent of *k*_a_ (see Section S2 and Figure S4 in Supplementary Materials).

Taken together, the results presented in this section show that in the same range of *k*_a_ from 0.005 $s^{-1}$ to 0.06 $s^{-1}$, Eg5-ΔTail motors cannot generate steady force to slide apart two antiparallel MTs whereas FL-Eg5 motors can generate the steady force. Moreover, the steady force generated by FL-Eg5 motors increases linearly with the increase of *k*_a_, namely the steady force increases linearly with the increase of the MT overlapping length for a given concentration of FL-Eg5 motors and increases linearly with the increase of FL-Eg5 concentrations for a given MT overlapping length. These results explain why FL-Eg5 rather than Eg5-ΔTail motors are used to slide apart two antiparallel MTs in cells.

## 3. Methods

### 3.1. Chemomechanical Coupling Pathway of Single Eg5-ΔTail

The model for the stepping of Eg5-ΔTail moving on a MT filament is the same as that for kinesin-1 proposed before [16,20], as schematically shown in Figure 1, where for clarity only the pair of heads that moves on the MT filament is shown. For convenience of reading, we re-describe briefly the model here (see [16,20] for detailed description). Throughout, we focus on saturating ATP concentrations.

We begin with the trailing head in ATP state binding strongly to MT-binding site I and the leading head in ATP state binding strongly to site II (Figure 1a). The trailing head with the forward NL orientation has a much larger rate of ATP transition to ADP than the leading head with the backward NL orientation (see discussion for parameter values in Section 2.1). Consider ATP transition to ADP in the trailing head, the head detaches easily from site I by overcoming the very weak affinity (*E*_w1_) to the local site I having large conformational changes induced by the strong interaction with ATP-head [34] and then diffuses to the intermediate (INT) position relative to MT-bound head with undocked NL, where the two heads have a high affinity [35] (Figure 1b). In INT state, the large conformational change of the head in ATP state occurs rapidly [36], reducing greatly the affinity between the two heads [35] and inducing the NL of the MT-bound head to dock [36]. Then, the detached head either (with probability *P*_E_) diffuses forward and binds to site III without the conformational changes with affinity *E*_w2_ (much larger than *E*_w1_) (Figure 1d) or (with probability 1 − *P*_E_) diffuses backward and binds to site I with affinity *E*_w2_ (Figure 1e) (noting that after the head transition to ADP the changed conformation of site I changes to the originally unperturbed one in time *t*_r_ of the order of 10 μs [37,38,39]). It is noted that the transition from Figure 1c to e requires undocking NL and induces the reverse large conformational change of the MT-bound head due to NL interference (see Refs. [16,20] for detailed discussion). After the detached ADP-head binding to MT, ADP is released (Figure 1a,f).

In Figure 1b, if the transition of ATP to ADP occurs before the great reduction of the high affinity between the two heads, the MT-bound head has the affinity *E*_w1_ to the local site II within the time period *t*_r_ (called Period I) (Figure 1g), during which the motor dissociates with a nearly 100% probability due to the pretty small *E*_w1_. In Figure 1d, if the transition of ATP to ADP in the trailing head occurs before the release of ADP from the leading head, the trailing head diffuses to INT position where the two heads have the high affinity and the MT-bound head has the affinity *E*_w2_ to site III (Figure 1h). During the period (called Period II) before ADP releasing from the MT-bound head, the motor has a large probability to dissociate by overcoming *E*_w2_. If the motor has not dissociated until ADP release from the MT-bound head, after ATP binding (Figure 1i) the system becomes the same state as that of Figure 1b except that the motor has moved a forward step. Similarly, in Figure 1e, if the transition of ATP to ADP in the leading head occurs before the release of ADP from the trailing head, the leading head will diffuse to INT position where the two heads have the high affinity and the MT-bound head has the affinity *E*_w2_ to site I (Figure 1j). During the period (Period II) before ADP release from the MT-bound head, the motor has a large probability to dissociate by overcoming *E*_w2_.

It is noted that in Figure 1a ATP transition to ADP in the leading head can also occur occasionally before ATP transition to ADP in the trailing head (not shown in Figure 1). Then, the leading head detaches from site II and diffuses to INT position. From INT position, the detached head can either (with probability *P*_E_) diffuse forward and re-bind to site II in time *t*_r_ or (with probability 1 − *P*_E_) diffuse backward and bind to the minus-end site next to site I. During these transitions, Period II can also occur.

### 3.2. Chemomechanical Coupling Pathway of Single FL-Eg5

The experimental data showed that the tail domain has a high affinity to ϕ-head, has a higher affinity to ADP-head than to ϕ-head, and has a very low affinity to ATP- or ADP.Pi-head [14]. These indicate that the rate of the tail releasing from ADP-head is very small (with a near-zero value), the rate of the tail releasing from ϕ-head is small (with a small value), and the rate of the tail releasing from ATP-head is very large, implying that the binding of the tail and that of ATP to the head are almost incompatible with each other. Thus, it is argued that only after the release of the tail from ϕ-head can ATP bind to the ϕ-head. Based on this argument and the chemomechanical coupling pathway of Eg5-ΔTail (Figure 1), the chemomechanical coupling pathway of FL-Eg5 at saturating ATP can be schematically shown in Figure 2, where for clarity the occurrence of the weak MT-binding periods (i.e., Period I and Period II) is not shown. The pathway is described as follows.

We begin with the trailing head (in ATP state) binding strongly to site II while the leading head (in ADP state and with tail bound to it) binding with weak affinity *E*_w2_ to site III (Figure 2a). Stimulated by MT, ADP is released rapidly from the leading head (Figure 2b). Then, either the tail releasing from the leading ϕ-head can occur before ATP transition to ADP in the trailing head, followed immediately by ATP binding to the leading head (Figure 2c), or the latter can occur before the former, with the trailing ADP-head moving to INT position and at the same time the tail binding to the detached ADP-head (Figure 2d). From Figure 2d, the tail is released from the MT-bound ϕ-head, followed immediately by ATP binding (Figure 2e). At INT state of Figure 2e, the large conformational change of the MT-bound head occurs rapidly, weakening the affinity between the two heads and inducing NL of the MT-bound head to dock (Figure 2f). Then, the detached ADP-head can either (with probability *P*_E_) diffuse forward and bind to site IV (Figure 2g) or (with probability 1 − *P*_E_) diffuse backward and bind to site II (Figure 2h). In Figure 2h, stimulated by MT, ADP is released rapidly from the trailing head (Figure 2i). Then, either the tail releasing from the trailing ϕ-head can occur before ATP transition to ADP in the leading head, followed immediately by ATP binding to the trailing head (Figure 2c), or the latter can occur before the former, with the leading ADP-head moving to INT position and at the same time the tail binding to the detached ADP-head (Figure 2j). From Figure 2j, the tail is released from the MT-bound ϕ-head, followed immediately by ATP binding (Figure 2k). At INT state of Figure 2k, the large conformational change of the MT-bound head occurs rapidly, weakening the affinity between the two heads and inducing NL of the MT-bound head to dock (Figure 2l). Then, the detached ADP-head can either (with probability *P*_E_) diffuse forward and bind to site III (Figure 2a) or (with probability 1 − *P*_E_) diffuse backward and bind to site I (Figure 2m). From Figure 2c if ATP transition to ADP occurs in the trailing head the system becomes the state of Figure 2e while if ATP transition to ADP occurs in the leading head the system becomes the state of Figure 2l.

## 4. Concluding Remarks

In this work, based on our previously proposed model for processive stepping of the single Eg5-ΔTail moving on MT and the prior biochemical data for the nucleotide-dependent interaction between the tail domain and head of Eg5, a model is proposed for processive stepping of the single FL-Eg5 moving on MT. For example, the experimental data indicating that the tail releases from ϕ-head with a low rate while from ATP-head is very large [14] implies that the binding of the tail and that of ATP to the ϕ-head are almost incompatible with each other. Thus, it is reasonably argued that only after the release of the tail from ϕ-head can ATP bind to the ϕ-head in the model. This leads to the deduction that the interaction of the tail with head slows ATPase activity even at saturating ATP. On the other hand, if it is simply argued that the tail binding to the ϕ-head induces the reduction of the second-order rate constant (*k*_bT_) of ATP binding, it is expected that at saturating ATP concentration (with a very large [ATP]) the time of ATP binding (1/(*k*_bT_[ATP])) would still be much shorter than the time of ATP hydrolysis and Pi release. This implies that at saturating ATP, with the tail the ATPase rate would be nearly the same as (or only slightly smaller than) that without the tail, which is inconsistent with the experimental data showing that the tail evidently slows the ATPase activity at saturating ATP [14]. The above discussion thus gives support to our argument and the model.

With the model the load dependences of velocity, ATPase rate, run length and dissociation rate for both the single Eg5-ΔTail and single FL-Eg5 are studied analytically, reproducing the available experimental data for the load dependences of velocity and run length for Eg5-ΔTail, reproducing the available experimental data for the load dependence of velocity for FL-Eg5 and explaining the experimental results about the effect of the tail on the ATPase rate and run length of the Eg5 motor under no load. Furthermore, with the determined parameter values for the single Eg5-ΔTail and FL-Eg5 motors, the force that can be generated by multiple Eg5-ΔTail and FL-Eg5 motors to slide apart two antiparallel MTs are studied numerically, with the numerical results being consistent with the available experimental data. The underlying mechanism is revealed of why the FL-Eg5 motors can generate the steady force whereas the Eg5-ΔTail motors cannot. This is due mainly to the interaction of the tail with the head modulating the chemomechanical coupling of the motor, which leads to the dissociation rate for FL-Eg5 before stall force having the catch-bond characteristic and the dissociation rate around the stall force for the FL-Eg5 being much smaller than that for the Eg5-ΔTail. In the future, it is hoped to test the predicted results on the load dependence of dissociation rate of the single FL-Eg5 from MT during the processive movement (Figure 4d).

## Figures and Tables

**Table 1:** Parameter values for Eg5-ΔTail and FL-Eg5.

Parameter	Eg5-ΔTail	FL-Eg5
*k*^(+)^ ($s^{-1}$)	12.8	12.8
*k*^(−)^ ($s^{-1}$)	*k*^(+)^/15	*k*^(+)^/15
*E*_D_ (*k*_B_*T*)	2.5	2.5
*k*_NL_ ($s^{-1}$)	200	200
*k*_D_ ($s^{-1}$)	50	50
$\delta_{w}$ (nm)	1	1
*d*^(+)^ (nm) *	2.2	2.2 and 8.2
*k*_w0_ ($s^{-1}$) *	20	5
*k*_r_ ($s^{-1}$)	–	4.6

Except that parameters with ‘*’ have different values, other parameters have the same values for Eg5-ΔTail and FL-Eg5. Symbol ‘–’ represents that the value is not required in the calculation.

## Data Availability

The data that support the findings of this study are available from the corresponding author upon reasonable request.

## Supplementary Materials

The following are available online at https://www.mdpi.com/article/10.3390/ijms22157857/s1.
