# Supplementary material for: Effect of Kinesin-5 Tail Domain on Motor Dynamics for Antiparallel Microtubule Sliding
Source: Int J Mol Sci. 2021 Jul 23;22(15):7857. doi: 10.3390/ijms22157857 (PMC8345995; doi:10.3390/ijms22157857)
Supplement: Supplementary file 1 [file ijms-22-07857-s001.zip › ijms-1253558-supplementary.pdf]

Supplemental Materials

Effect of kinesin-5 tail domain on motor dynamics for antiparallel microtubule sliding

Supplemental text

S1. Monte-Carlo simulations

In our simulations, we take time step $h = 10^{-4}$ s. For each kinesin-5 tetramer bound to the overlapping MTs, we take 7 independent random numbers uniformly distributed between 0 and 1, *ran*1, *ran*2, … *ran*7. During each time step h, if *ran*1 < εh the pair of heads bound to a MT would detach from the MT. If *ran*2 < εh the other pair of heads bound to the other MT would detach from the MT. If *ran*3 < $k_F h$ the pair of heads bound to a MT would take a forward step, and if *ran*4 < $k_B h$ the same pair of heads would take a backward step. If *ran*5 < $k_F h$ the other pair of heads bound to the other MT would take a forward step, and if *ran*6 < $k_B h$ the same pair of heads would take a backward step. When only one pair of heads is bound to a MT, if *ran*7 < $\mu_5 h$ the other detached pair of heads would rebind to the other MT. When two pairs of head are simultaneously detached from MTs, the tetramer would dissociate into solution. In addition, we take another random number uniformly distributed between 0 and 1, *ran*8. If *ran*8 < $k_a h$, a kinesin-5 tetramer in the solution wound bind to one of the overlapping MTs. Note that the detaching, rebinding, forward stepping, backward stepping and binding from solution of one kinesin-5 tetramer are independent with those of another one.

S2. Simulations of Eg5 movement within MT sliding zones

To simulate Eg5 movement within MT overlapping region, we fix one MT and keep the other antiparallel MT free, as done in the experiments of Bodrug et al. [S2]. No external load acts on the free MT. The simulation procedure is described as follows.

We take initially ten kinesin-5 tetramers bound to the overlapping MTs. Each pair of heads of the tetramer can detach, step forward, step backward and rebind to MT, which can be simulated with the Monte-Carlo simulation algorithm as described in Section S1. Note that the detaching rate, forward stepping and backward stepping depend only on the force arising from the stretching of the stalk, because no external

force is present. We denote by n_1 the net number of steps that the pair of heads bound to the fixed MT takes within time period h and by n_2 the net number of steps that the other pair of heads bound to the free MT takes within time period h, where n_1 and n_2 are positive when the pair of heads moves towards the plus end of the MT. The displacement of the free MT within time period h can then be written as $z=(n_1+n_2)d/N_2$, where d = 8.2 nm is the step size, N_2 is the total number of kinesin-5 motors with the two pairs of heads bound simultaneously to the overlapping MTs, and z is defined to be positive along the plus-end direction of the fixed MT. Denote by $x_1^{(i)}(t)$ and $x_2^{(i)}(t)$ the center-of-mass positions of the pair of heads of motor i bound to the fixed MT and bound to the free MT at moment t, respectively. The force, which arises from the stretching of the stalk, on the two pairs of heads can be calculated with $F_i=K_5\left|x_1^{(i)}(t)-x_2^{(i)}(t)\right|$. When the two pairs of heads of motor i are bound to the two MTs, after a time period h, $x_1^{(i)}(t+h)=x_1^{(i)}(t)+n_1d$ and $x_2^{(i)}(t+h)=x_2^{(i)}(t)-n_2d+z$. When the pair of heads is detached from the free MT, after a time period h, $x_1^{(i)}(t+h)=x_1^{(i)}(t)+n_1d$ and $x_2^{(i)}(t+h)=x_1^{(i)}(t+h)$. When the pair of heads is detached from the fixed MT, after a time period h, $x_2^{(i)}(t+h)=x_2^{(i)}(t)-n_2d+z$ and $x_1^{(i)}(t+h)=x_2^{(i)}(t+h)$. Since when the two pairs of heads of a motor are simultaneously detached from the two MTs the motor is dissociated into solution, the position of the motor is recorded no longer. With the above simulations, the displacement of the center-of-mass position of a motor within the MT overlap can be calculated. Similarly, the displacement of the free MT can also be calculated.

In Figure S3a we show some trajectories for the displacement of the single Eg5-ΔTail motors within the MT overlap. In Figure S3b, we show some trajectories for the displacement of the single FL-Eg5 motors within the MT overlap. As mentioned above, in Figure S3 when the motor is dissociated into solution, i.e., when its two pairs of heads are simultaneously detached from the two MTs, the position of the motor is recorded no longer. From Figure S3, we see firstly that an Eg5-ΔTail motor can move within the overlap only for a short time whereas a FL-Eg5 can move within the overlap for a long time. This is due to the fact that the Eg5-ΔTail motor has a larger dissociation rate from MT and a smaller rebinding rate to MT than the FL-Eg5. Secondly, we see that the Eg5-ΔTail motor shows bi-directional movement with frequent directional

reversals whereas the FL-Eg5 motor shows unidirectional movement with infrequent directional reversals. These features for the two motors resemble well the experimental data of Bodrug et al. [S2]. These features are also due to that the Eg5-ΔTail motor has a larger dissociation rate from MT, a larger velocity and a smaller rebinding rate to MT than the FL-Eg5. In Figure 4a we show some trajectories for the displacement of the free MT by multiple FL-Eg5 motors. The free MT shows unidirectional movement with rare directional reversals (Figure 4a), with the velocity being nearly independent of k_a (Figure 4b), implying that the velocity is nearly independent of the motor number.

References

[S1] Singh S.K., Pandey H., Al-Bassam J., Gheber L. (2018) Bidirectional motility of kinesin-5 motor proteins: structural determinants, cumulative functions and physiological roles. Cell. Mol. Life Sci. 75, 1757–1771.

[S2] Bodrug T., Wilson-Kubalek E.M., Nithianantham S., Thompson A.F, Alfieri A., Gaska I., Major J., Debs G., Inagaki S., Gutierrez P., Gheber L., McKenney R.J., Sindelar C.V., Milligan R., Stumpff J., Rosenfeld S.S, Forth S.T., Al-Bassam J. (2020) The kinesin-5 tail domain directly modulates the mechanochemical cycle of the motor domain for anti-parallel microtubule sliding. eLife 9, e51131.

Supplemental figures

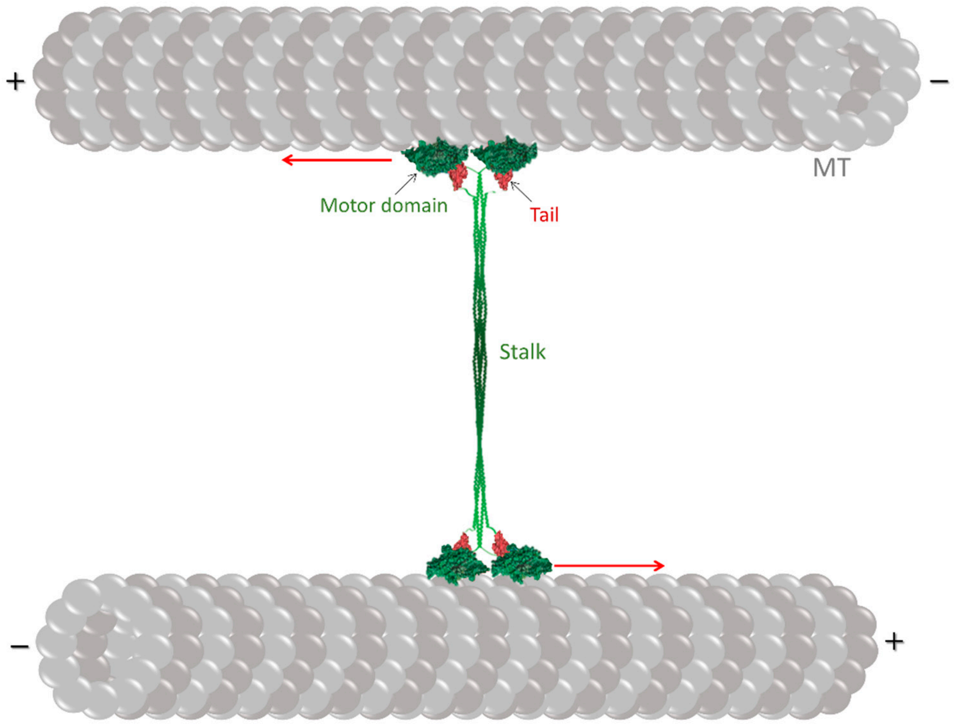

Figure S1. Schematic diagram of kinesin-5 homotetramer bound to two antiparallel MTs (adapted from Singh et al. [S1]). The tetramer is composed of two pairs of N-terminal motor domains (or heads) and two pairs of C-terminal tail domains, which are situated at the opposite ends of a common stalk. One pair of heads of the tetramer binds to one MT and the other pair binds to another antiparallel MT, crosslinking the two MTs. Powered by the hydrolysis of ATP the two pairs of heads can move on the two antiparallel MTs independently, sliding apart the two MTs. Red arrows indicate the plus-end direction for the movement of a pair of heads on the MTs.

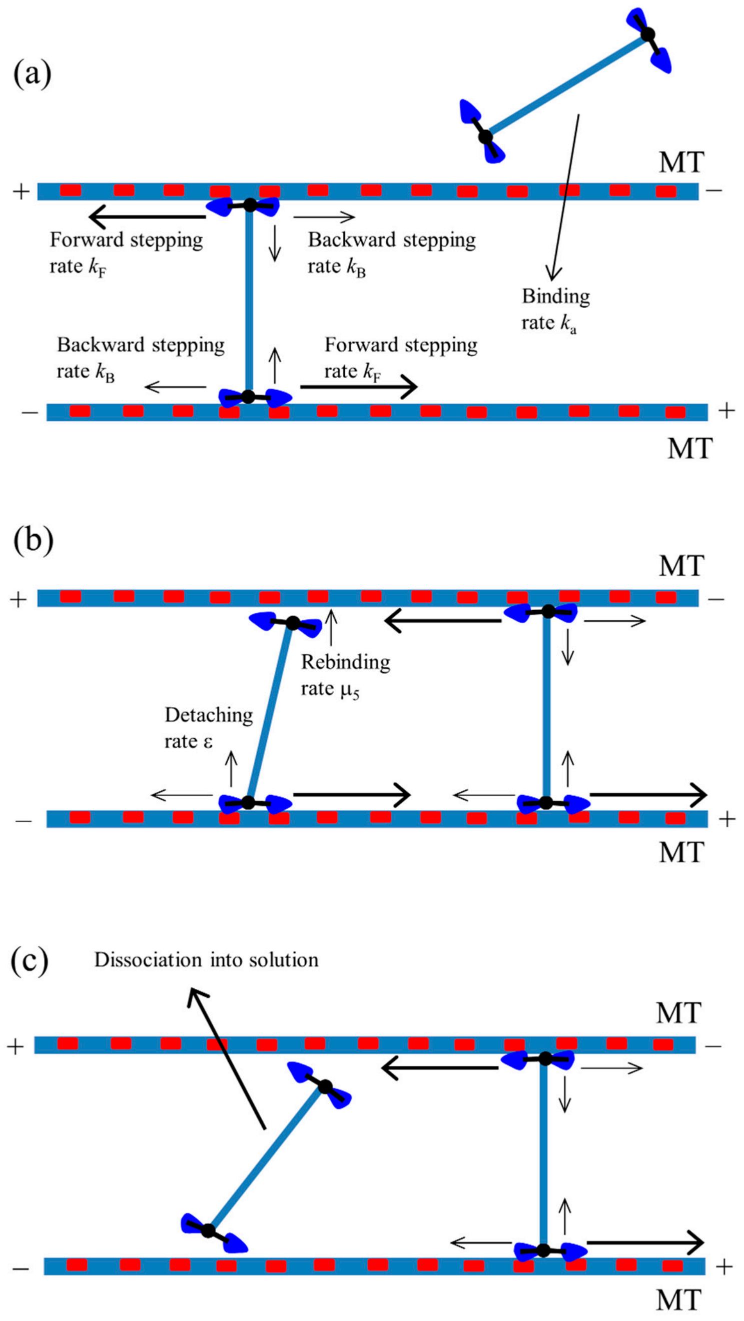

Figure S2. Schematic illustrations of kinesin-5 activities during MT sliding in the simulation. **(a)** A kinesin-5 tetramer (right) in solution can bind to the overlapping MTs with rate k_a. The two pairs of heads of another kinesin-5 tetramer (left) bound to the two MTs can independently step forward with rate k_F, step backward with rate k_B and detach with rate ε. **(b)** A pair of heads of a kinesin-5 tetramer (left) bound to one MT can step forward with rate k_F, step backward with rate k_B and detach with rate ε, while the other pair of heads of the tetramer detached from the other MT can rebind to the MT with rate μ_5. **(c)** When two pairs of heads are simultaneously detached from MTs the kinesin-5 tetramer (left) dissociates into solution.

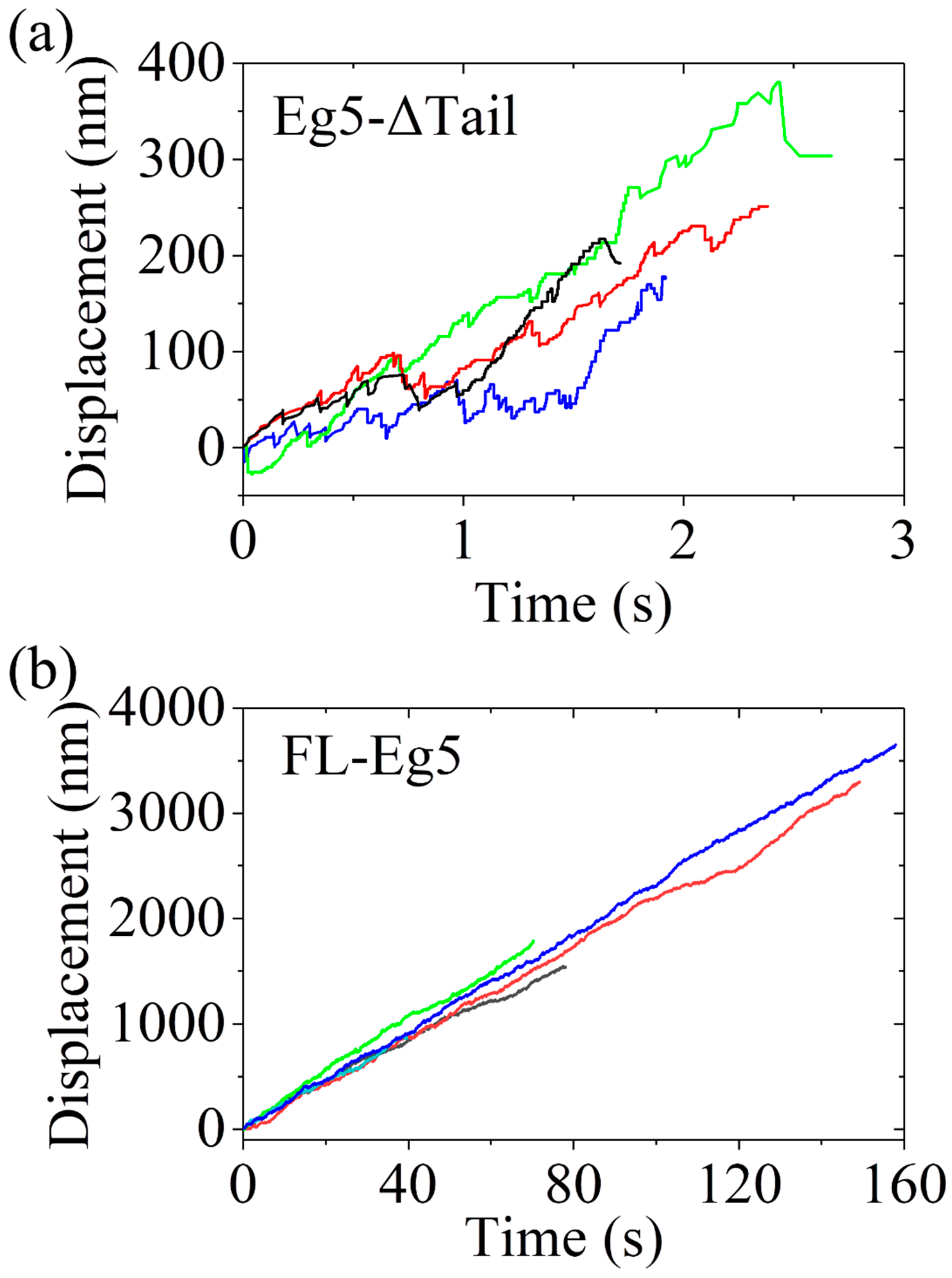

Figure S3. Movement of the single motor within MT overlapping zone during MT sliding by multiple motors. One MT is fixed and the other antiparallel MT is free. The parameter values are the same as those used in Figs. 5 and 6. **(a)** Displacement trajectory of a motor within overlapping MTs driven by multiple Eg5-ΔTail motors. Curves of different colors correspond to different motors in one simulation realization. **(b)** Displacement trajectory of a motor within overlapping MTs driven by multiple FL-Eg5 motors. Curves of different colors correspond to different motors in one simulation realization.

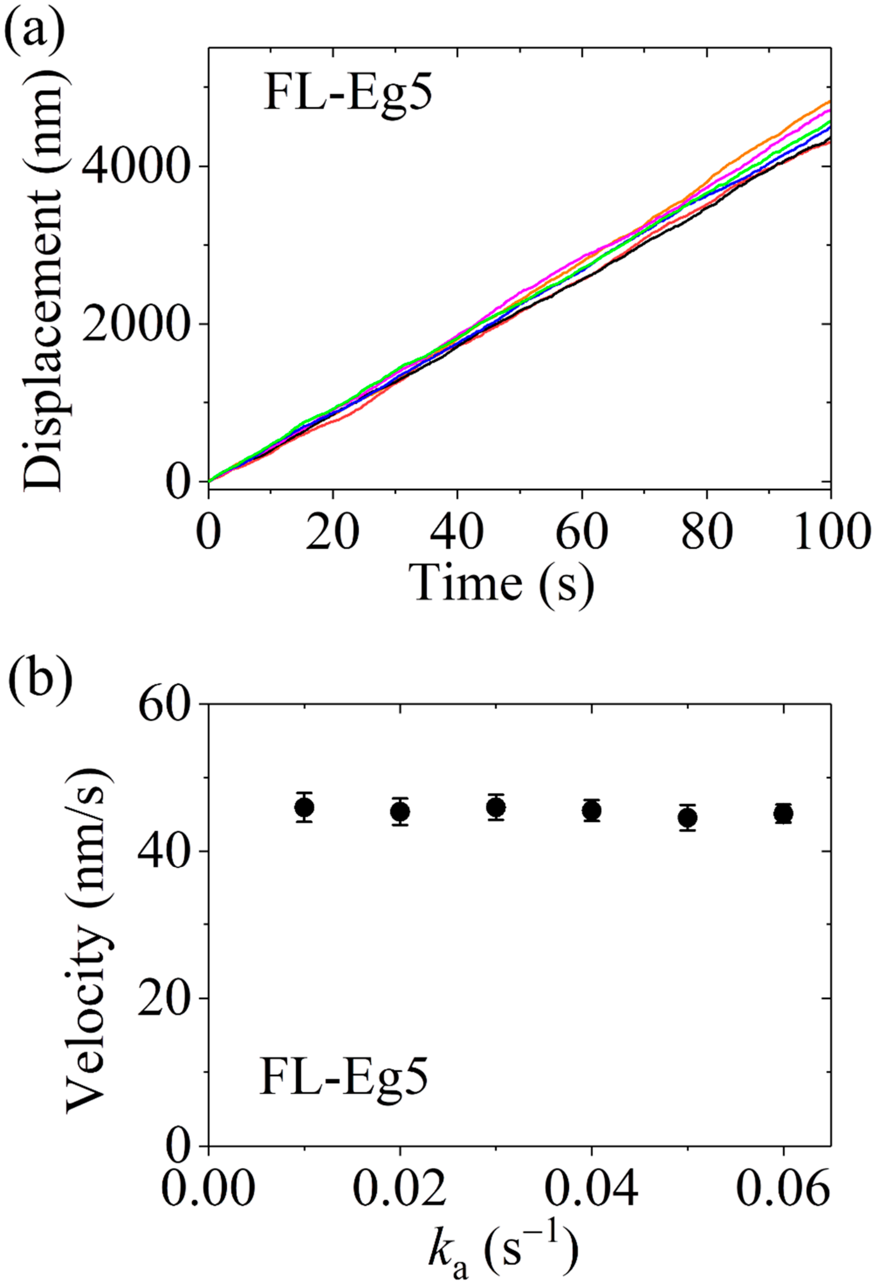

Figure S4. Movement of free MT during MT sliding by multiple FL-Eg5 motors. One MT is fixed and the other antiparallel MT is free. Parameter values are the same as those used in Figs. 5 and 6. **(a)** Displacement trajectory of the free MT, with $k_a = 0.03$ s^{-1} . Curves of different colors correspond to different, independent simulation realizations. **(b)** MT sliding velocity versus k_a. The velocity and the standard deviation at a given k_a are calculated from 20 trajectories that are simulated with 20 independent realizations.
